# Use of Propensity Score Matching to Compare Short Outcomes from Transoral and External Surgical Approaches in Patients with Deep-Lobe Parotid Pleomorphic Adenomas

**DOI:** 10.3390/curroncol28040272

**Published:** 2021-08-18

**Authors:** Yue Fan, Shuguang Li, Shuting Yu, Xiaoli Zhu, Xiaohua Shi, Wuyi Li, Zhiqiang Gao, Xingming Chen

**Affiliations:** 1 Department of Otolaryngology-Head and Neck Surgery, Peking Union Medical College Hospital, Chinese Academy of Medical Science and Peking Union Medical College, Beijing 100730, China; chromefan@126.com (Y.F.); shuguang1002@163.com (S.L.); yushuting@pumch.cn (S.Y.); zhuxlent@sina.com (X.Z.); wuyilient@126.com (W.L.); dr_talllee@sina.com (Z.G.); 2 State Key Laboratory of Complex Severe and Rare Diseases, Peking Union Medical College Hospital, Chinese Academy of Medical Science and Peking Union Medical College, Beijing 100730, China; xiaohuashi82@163.com; 3 Department of Pathology, Peking Union Medical College Hospital, Chinese Academy of Medical Science and Peking Union Medical College, Beijing 100730, China

**Keywords:** propensity score matching, pleomorphic adenoma, parotid gland, deep lobe, transoral approach, recurrence

## Abstract

To compare the outcomes of patients who had deep-lobe parotid gland pleomorphic adenomas (PAs) that extended into the parapharyngeal space after surgical treatment, using a transoral approach or an external approach. One hundred and twelve eligible patients, with deep-lobe parotid gland PAs, were enrolled in this retrospective study. The surgical outcomes were compared for patients who received a transoral approach and an external approach, using 1:1 propensity score matching (PSM). The outcome measures were recurrence rate, facial nerve deficit, Frey’s syndrome, and hospitalization time. The median follow-up time was 4.8 years. After PSM, the transoral approach and external approach groups had no statistically significant difference in recurrence (10.3% vs. 3.4%; *p* = 0.201). The transoral approach group had no facial nerve deficit, but 5 of 29 patients (17.2%) in the external approach group had transient facial nerve paralysis (*p* = 0.052). The external approach group had a longer hospitalization time than the transoral approach group (5 vs. 4 days, *p* = 0.0017). The use of a transoral surgical approach to treat patients with deep-lobe parotid gland PAs led to low recurrence, shorter hospitalization times, and good functional and cosmetic outcomes.

## 1. Introduction

Pleomorphic adenoma (PA) is the most common tumor of the parotid gland, and accounts for approximately 60 to 80% of all benign parotid gland tumors [1]. Most of these tumors originate from the superficial lobe, and 10 to 12% arise from the deep lobe [2,3]. A PA located in the deep lobe usually extends into the parapharyngeal space (PPS) and presents as an asymptomatic mass that can be unnoticed for a long time.

The PPS is a deep potential neck space that is medial to the parotid space, has an inverted pyramid shape, and extends from the base of the skull to the hyoid bone. Surgeons may use a variety of approaches (transoral, transcervical, transcervical–transparotid, transparotid, and transmandibular) for thxe management of PPS tumors [4,5,6,7]. As the overall trend in surgery moves towards minimally invasive techniques, many surgeons now favor a transoral approach, and there is evidence that transoral surgery, for the resection of benign lesions within the PPS, provides good results [8,9,10,11,12].

For a PA in the deep lobe of parotid gland that extends into the PPS, a transoral approach provides a direct route to the tumor. Thus, many surgeons reported favorable results from the use of the transoral method, with or without assistance from an endoscope or robot [12,13]. However, there is still debate about the use of the transoral approach. Some surgeons considered this approach as questionable, because of the poor visualization, lack of control over the great vessels and facial nerve, and the risk of tumor fragmentation and capsular breach, which may lead to recurrence [5].

We conducted a retrospective study of a large number of patients with deep-lobe parotid PAs, who received surgery by an external approach or a transoral approach at a single large institution, and then compared the surgical outcomes, especially the recurrence rates. To reduce potential selection bias, caused by the observational nonrandomized study design, we implemented propensity score matching (PSM), to achieve a more balanced study cohort. To the best of our knowledge, this is the first study to employ a PSM design to control for variables of known prognostic significance, in a comparison of different surgical approaches for PA of the parotid.

## 2. Materials and Methods

### 2.1. Patient Population

This retrospective study examined the records of 112 patients at Peking Union Medical College Hospital. Each patient presented with a PA in the deep lobe of the parotid gland from January 2000 to October 2018. The study protocol was approved by the Ethics Committee of Peking Union Medical College Hospital. The inclusion criteria were pathologically confirmed, previously untreated PA of the deep lobe of the parotid gland and receipt of radical resection; and post-surgical follow-up time of at least 2 years. The exclusion criteria were deep-lobe parotid tumor without parapharyngeal space involvement; dumbbell-shaped tumor from the deep lobe of the parotid gland; suspicion of malignant PA before the operation or confirmation of malignancy by pathology after the operation; and post-surgical follow-up time less than 2 years.

Clinical information was collected to analyze the impact of patient age, sex, radiological characteristics of tumor, histopathology, surgical approach, and surgical outcome. The average longest dimension of each tumor was recorded based on pre-operative computed tomography (CT) or magnetic resonance imaging (MRI). Patients were observed with a follow-up of every two years by physical examination and CT/MRI. The primary outcome was recurrence rate and the secondary outcomes were complications.

### 2.2. Surgical Procedure

Patients received surgery using a transoral, transcervical, transparotid, or transcervical–transparotid approach. All tumors were completely excised.

For the transoral approach, a Crowe–Davis retractor was placed to open the mouth and retract the tongue. A vertical curvilinear incision was made along the bulge on the mucosa of the soft palate and tonsillar pillar. The mucosa, submucosa, and constrictor muscle over the tumor were divided into the tumor capsule. Blunt dissection commenced on the tumor capsule at the superior aspect of the mass and proceeded inferiorly. The posterior and lateral margin of the tumor was dissected last, by use of a blunt instrument or finger dissection. The dissection was further assisted by 0° and 30° endoscope as needed. To ensure the tumor was completely removed without remnants, a part of the parotid gland tissue around the pedicle was also removed. The wound was irrigated and hemostasis was performed. The wound was closed in layers, and an intraoral drain was placed. Nasogastric tube was inserted at the end of the procedure. The drainage was removed after 3 to 5 days, and nasogastric tube was removed after 5 days and patients were started on liquid diet.

The other three external approach operations were performed using conventional surgical techniques [7,14,15].

### 2.3. Statistical Analysis

Continuous data were presented as mean ± SD or medians with interquartile ranges (IQR), whereas categorical data were presented as a percentage. Variables were compared using t-test for continuous variables and chi-square test for categorical variables. Univariate Cox analysis and log-rank test were used for qualitative and quantitative variables to determine whether there is significant difference between the transoral approach and external approach groups. The adjusted HR and 95% CI were calculated. Covariates with *p* < 0.1 in the above analysis and the available literature were selected for the multivariate Cox proportional hazard analysis. Multivariate Cox regression analysis was adopted to assess the independent association between recurrence and surgical approach. Several models were performed to conform this association. We adjusted age and sex in model I, and age, sex, tumor size, capsule disruption and fragmentation in model II.

An extended Cox model approach was used for different covariates adjusted models. To generate propensity scores, we used a Cox proportional hazards regression model using a 1:1 nearest-neighbor matching algorithm with no replacement and a caliper width of 0.2 of the standard deviation of the logit of the propensity score. The covariates were age, tumor size, capsule disruption and fragmentation. To compare baseline variables before and after matching, standardized mean differences (SMDs) were calculated.

The log-rank test was used to compare Kaplan–Meier curves of recurrence-free survival in the transoral approach and external approach groups. Time to recurrence time was calculated from date of surgery to date of recurrence as recorded. Censored observation were defined as patients who did not have recurrence; the censoring time was calculated from date of surgery to the last assessment or loss to follow-up.

All tests were 2-sided, and *p*-value < 0.05 was considered significant. An SMD > 0.1 was considered significant. All the analyses were performed with the statistical software packages R (http://www.R-project.org, The R Foundation, Version: 3.6.3, access on 12 February 2020).

## 3. Results

### 3.1. Baseline Characteristics

We retrospectively enrolled 112 patients (58 male and 54 female), from January 2000 to October 2018. The mean age was 49.1 ± 10.2 years old, ranging from 27 to 74 years old. Thirty-six patients (32.1%) received a transoral approach, 29 patients (25.9%) received a transcervical approach, 24 patients (21.4%) received a transparotid approach, and 23 patients (20.5%) received a transcervical–transparotid approach. Overall, the average longest dimension of the tumor was 3.6 cm, based on preoperative CT/MRI. Capsule disruption, according to the pathological report, was noted in 35 cases (31.2%), while tumor fragmentation during operation was observed in 13 cases (11.6%). The characteristics of the cohort are summarized in Table 1.

The univariate Cox proportional hazards regression analyses indicated that a different surgical approach was not a potential prognostic factor for recurrence. The tumor size, capsule disruption, and fragmentation were significantly associated with the recurrence rate (Table 2).

Before PSM, the external approach group was older, and had large tumors and a higher capsule disruption rate. The fragmentation rate was higher in the transoral approach group. We used covariates with *p* < 0.1 in Table 2, and age for the 1:1 PSM. Thus, we matched the 29 patients who underwent transoral approach surgery with 29 patients who underwent external approach surgery. As shown in Table 1, the covariates of the matched cohorts were balanced between the two groups (SMD < 0.1).

### 3.2. Surgical Outcomes

**Figure 1:**
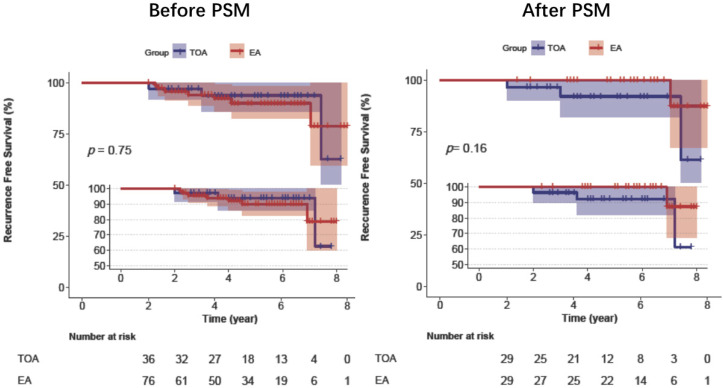
Kaplan–Meier analysis of recurrence-free survival of patients in the transoral approach and external approach groups before and after PSM. Abbreviations: TOA, transoral approach; EA, external approach; PSM, propensity score matching.

The median follow-up time for the whole cohort was 4.8 years. Before PSM, the recurrence rates of the transoral and external approach groups were 8.3% and 9.2%, and the difference was not significant (*p* = 0.754). After adjustment in the multivariate analysis of the original cohort, the patients who underwent transoral approaches did not have an increased recurrence rate. After PSM, the percentage of recurrences in the transoral approach cohort was 10.3%, and that in the external approach cohort was 3.4% (*p* = 0.201) (Table 3). Kaplan–Meier analysis confirmed that the two groups had no significant difference in recurrence before and after PSM (Figure 1).

### 3.3. Complications and Duration of Hospitalization

The total hospital stay after surgery was longer in the external approach group in the original cohort (*p* < 0.001), and in the matched cohort, the patients who underwent the transoral approach had one day less hospital stay compared to the external approach group (*p* = 0.0017) (Table 4).

No patient presented with secondary hemorrhage. Before PSM, postoperative transient facial nerve complications were present in 18 of the 76 patients (23.7%) in the external approach group, but present in no patients in the transoral approach group (*p* = 0.004). Recovery from facial nerve paralysis occurred after a maximum of 12 weeks. After PSM, there was no significant difference in the occurrence rate of transient facial nerve paralysis between the two groups (*p* = 0.052). Only two patients reported Frey’s syndrome in the original cohort, and there was no difference in the incidence of Frey’s syndrome between the two groups after PSM (Table 4).

## 4. Discussion

Surgical excision is the primary treatment for PA of the parotid gland. Resection of a PA in the deep lobe, with invasion of the PPS, is difficult, due to its depth and proximity to vital neurovascular structures. The goals of surgical resection are complete tumor removal, preservation of function, minimal morbidity, and satisfactory aesthetic outcome. The literature describes many different surgical approaches, and these can be classified as transoral or external [5,16,17].

The choice of surgical approach is usually determined by the tumor size, location, vascularity, and the patients/surgeons’ preference. A major consideration is wide intra-operative visibility for safe radical dissection and minimal functional or cosmetic after-effects [5,6]. Each surgical approach has its proponents. Among the external approaches, some surgeons consider the transcervical approach as best for tumors originating from the deep lobe of the parotid gland [18]; some surgeons commonly use a transparotid approach for resecting deep-lobe parotid gland tumors, especially dumbbell-shaped lesions [19]; and some surgeons consider the transcervical–transparotid approach in patients with prestyloid tumors that have broad attachment to the deep lobe of the parotid gland [20]. The common disadvantage of all external approaches is that they can lead to facial and or cervical scars. Because exposure and resection of PAs of the deep lobe of the parotid gland are relatively difficult, some surgeons may use superficial parotidectomy, with full dissection of the facial nerve, but this can lead to facial nerve deficits [21].

Use of a transoral surgical approach for these tumors is not limited by some of the constraints of the external approach. For example, the transoral approach provides direct access to the PPS and has obvious advantages for the cosmetic outcome. Nonetheless, some surgeons believe that this approach is unsafe, because it does not provide good control of the vascular and nervous structures, due to the small working area, and that it does not provide adequate exposure for removal of the tumor, which is often extirpated as fragments, thus possibly increasing the rate of recurrence [7,11,22,23]. Because of this controversy, we implemented multivariate Cox analysis and PSM for patients who received a transoral approach or an external approach, and to compare their recurrence rates, incidence of complications, and hospitalization times. We used age, tumor size, capsule disruption, and fragmentation as the covariates.

One of the first studies to assess the efficacy of the transoral approach was published in 1988. In this study, Goodwin and Chandler reported a 25% recurrence rate following transoral resection of PAs after a 5-year follow-up [15]. A 2009 review reported an overall recurrence rate of only 8% among 35 patients with PAs and extension into the PPS [16]. Our results showed that the recurrence rate of the transoral approach group was 8.3% before PSM and 10.3% after PSM (*p* = 0.754); the recurrence rate of the external approach group was 9.2% before PSM and 3.4% after PSM (*p* = 0.201), but these differences were not significant.

Some surgeons advocated a transoral surgical approach for benign prestyloid space tumors with diameters less than 3 cm [7,22,24,25]. The average tumor diameter in our transoral approach group was 3.20 ± 0.84 cm, and complete excision was achieved in all the patients. We believe that experienced surgeons are able to completely resect tumors using a transoral approach, although limited exposure may have led to the tumor decompressing during surgery, especially for tumors with diameters more than 4 cm. There is concern about the possible seeding of PAs when they are removed in fragments. In the present study, the longest tumor dimension in two of the three patients who had recurrence was over 5 cm, and our univariate analysis showed that a large tumor size was significantly associated with a greater risk of recurrence. When considering the transoral surgical approach, we therefore suggest caution when the tumor is large. In addition, we also suggest that a small piece of parotid tissue, around the pedicle of the tumor, should be excised with the tumor, to ensure complete extracapsular resection. Endoscopy could help, by providing magnification, illumination, and an improved operational view. With endoscopic assistance, residual tumor tissue can be more easily detected and removed.

Since 2007, more and more surgeons began to use trans-oral robotic surgery (TORS) for the management of PPS tumors [8,9,26]. The improved fine motor control, in addition to the tremor-free movement and three-dimensional binocular vision offered by TORS, leads to a dissection that is more precise than conventional transoral approaches, which significantly expanded surgical indications [27]. TORS could reduce the risk of tumor rupture, incomplete removal, and unmanageable complications during the operation, and had been proven to be safe and feasible in the treatment of PPS tumors. In this study, we believed that dumbbell-shaped PAs are not suitable for transoral surgery, due to the limited space available for tumor removal and the difficulty in protecting the facial nerve. However, Ansarin and Smriti reported tumors originating from the retrostyloid compartment that were successfully managed with TORS [8,28]. Arshad also indicated that in the case of retrostyloid tumors, TORS is not a contraindication, as carotids would be displaced anterolateral to the tumor [29]. It is suggested that TORS could expand the transoral corridor.

The most common complication in our patients was facial nerve paralysis. Historically, the rate of facial nerve injury, following surgery of a primary deep-lobe tumor of the parotid gland, was 6.5% [30]. In our external approach group, 18 patients experienced transient facial nerve paralysis of HB grade II–IV. Especially for larger tumors, the anatomical preservation of the facial nerve does not ensure functional preservation. Although preservation of the facial nerve was achieved in all the patients in the transoral approach group, there were no formal attempts to identify the facial nerves. Moreover, 22 patients (75.86%) in the matched transoral approach group were discharged home within 4 days, but the external approach group had a significantly longer hospitalization time.

One major drawback, aside from the known limitations of retrospective analysis, is the rather short follow-up time. At our institution, the transoral approach was not performed until 2010, and was applied more frequently in recent years. Thus, the follow-up period of the transoral approach group was not long enough for some patients to attain reliable oncological outcomes. Future long-term follow-up investigations are required, to assess the long-term recurrence rate, especially for evaluating the real impact of tumor spillage on the recurrence rate. Another drawback is that we failed to add the histologic subtypes of PAs and the presence of pseudopodia as variables. Moreover, since the choice of the transoral approach or the external approach is highly influenced by the surgeons’ preference and experience to a considerable extent, the different surgeons may had biased the prognosis in this study.

## 5. Conclusions

The present PSM study found no significant difference in the recurrence rate of parotid PAs invading the PPS in patients who received a transoral surgical approach or an external surgical approach. A benefit of the transoral approach is that it may provide more efficient facial nerve preservation. With the advent of more minimally invasive techniques, including robotic surgery, a transoral surgical approach should be considered for more patients with PAs in the deep lobe of the parotid gland.

## Figures and Tables

**Table 1:** Baseline characteristics of patients in the transoral approach and external approach groups before and after PSM.

Variables	Original Cohort			Matched Cohort		
	TOA	EA	SMD	TOA	EA	SMD
*n*	36	76		29	29	
Age, Mean ± SD	46.44 ± 11.54	50.38 ± 9.26	0.376	47.62 ± 12.00	47.86 ± 9.25	0.023
Tumor size, Mean ± SD	3.20 ± 0.84	3.92 ± 1.12	0.731	3.20 ± 0.80	3.21 ± 0.86	0.012
Capsule disruption, *n* (%)			0.203			<0.001
No	27 (75)	50 (65.8)		22 (75.9)	22 (75.9)	
Yes	9 (25)	26 (34.2)		7 (24.1)	7 (24.1)	
Fragmentation, *n* (%)			0.224			<0.001
No	30 (83.3)	69 (90.8)		27 (93.1)	27 (93.1)	
Yes	6 (16.7)	7 (9.2)		2 (6.9)	2 (6.9)	

Abbreviations: TOA, transoral approach; EA, external approach; SMD, standardized mean differences.

**Table 2:** Univariate Cox proportional hazards models evaluating the association between the variables and recurrence rate before PSM.

Variables	HR	95%CI	*p*-Value
Surgical approaches			0.754
EA	Reference		
TOA	1.24	0.32, 4.81	
Age	1.02	0.96, 1.09	0.483
Sex			0.182
Female	Reference		
Male	2.52	0.65, 9.8	
Tumor size	3.18	1.8, 5.62	<0.001
Capsule disruption			0.009
No	Reference		
Yes	7.9	1.67, 37.3	
Fragmentation			0.002
No	Reference		
Yes	8.11	2.16, 30.44	

Abbreviations: EA, external approach; TOA, transoral approach; F, female; M, male; HR, hazard ratio; CI, confidence interval.

**Table 3:** Association between surgical approaches and recurrence before and after PSM.

	Original Cohort							Matched Cohort		
	**Recurrence**	**Non-Adjusted**		**Adjust I**		**Adjust II**		**Recurrence**		
	**(%)**	**HR (95%CI)**	***p*-Value**	**HR (95%CI)**	***p*-Value**	**HR (95%CI)**	***p*-Value**	**(%)**	**HR (95%CI)**	***p*-Value**
TOA	3 (8.3)	Reference		Reference		Reference		3 (10.3)	Reference	
EA	7 (9.2)	1.24 (0.32, 4.81)	0.754	1.18 (0.3, 4.58)	0.811	0.18 (0.02, 1.41)	0.103	1 (3.4)	0.22 (0.02, 2.22)	0.201

Notes: Adjust I model adjusts for age and sex; adjust II model adjusts for adjust I + tumor size, capsule disruption and fragmentation. Abbreviations: EA, external approach; TOA, transoral approach; HR, hazard ratio; CI, confidence interval.

**Table 4:** The total hospital stays and complications of the transoral approach and external approach groups before and after PSM.

Outcomes	Original Cohort			Matched Cohort		
	TOA	EA	*p*-Value	TOA	EA	*p*-Value
Hospitalization, Median (IQR)	4.0 (3.0, 4.2)	5.0 (5.0, 6.0)	<0.001	4.0 (3.0, 4.5)	5.0 (4.0, 5.0)	0.0017
Complications, n (%)				3.20 ± 0.80	3.21 ± 0.86	
Transient facial paralysis			0.004			0.052
No	36 (100)	58 (76.3)		29 (100)	24 (82.8)	
Yes	0 (0)	18 (23.7)		0 (0)	5 (17.2)	
Frey’s Syndrome			1			1
No	36 (100)	74 (97.4)		29 (100)	29 (100)	
Yes	0 (0)	2 (2.6)		0 (0)	0 (0)	

## Data Availability

The data presented in this study are available in Result part.
